# Multi-Task Water Quality Colorimetric Detection Method Based on Deep Learning

**DOI:** 10.3390/s24227345

**Published:** 2024-11-18

**Authors:** Shenlan Zhang, Shaojie Wu, Liqiang Chen, Pengxin Guo, Xincheng Jiang, Hongcheng Pan, Yuhong Li

**Affiliations:** 1 Key Laboratory of Advanced Manufacturing and Automation Technology, Education Department of Guangxi Zhuang Autonomous Region, Guilin University of Technology, Guilin 541006, China; zsl@glut.edu.cn (S.Z.); wrmcake@163.com (S.W.); clqssg@163.com (L.C.); 2120231331@glut.edu.cn (P.G.); 2120211105@glut.edu.cn (X.J.); 2 College of Mechanical and Control Engineering, Guilin University of Technology, Guilin 541006, China; 3 College of Environment and Science, Guilin University of Technology, Guilin 541006, China; 4 Guilin Center for Agricultural Science & Technology Research, Guilin 541006, China

**Keywords:** water quality detection, deep learning, colorimetric sensor

## Abstract

The colorimetric method, due to its rapid and low-cost characteristics, demonstrates a wide range of application prospects in on-site water quality testing. Current research on colorimetric detection using deep learning algorithms predominantly focuses on single-target classification. To address this limitation, we propose a multi-task water quality colorimetric detection method based on YOLOv8n, leveraging deep learning techniques to achieve a fully automated process of “image input and result output”. Initially, we constructed a dataset that encompasses colorimetric sensor data under varying lighting conditions to enhance model generalization. Subsequently, to effectively improve detection accuracy while reducing model parameters and computational load, we implemented several improvements to the deep learning algorithm, including the MGFF (Multi-Scale Grouped Feature Fusion) module, the LSKA-SPPF (Large Separable Kernel Attention-Spatial Pyramid Pooling-Fast) module, and the GNDCDH (Group Norm Detail Convolution Detection Head). Experimental results demonstrate that the optimized deep learning algorithm excels in precision (96.4%), recall (96.2%), and mAP50 (98.3), significantly outperforming other mainstream models. Furthermore, compared to YOLOv8n, the parameter count and computational load were reduced by 25.8% and 25.6%, respectively. Additionally, precision improved by 2.8%, recall increased by 3.5%, mAP50 enhanced by 2%, and mAP95 rose by 1.9%. These results affirm the substantial potential of our proposed method for rapid on-site water quality detection, offering new technological insights for future water quality monitoring.

## 1. Introduction

Due to the development of human society, the process of large-scale urbanization and industrialization has had harmful effects on the water bodies in the environment. The pollution of water resources generally causes adverse effects on animal, plant, and human life. Harmful pollutants in water enter the human body through drinking water and threaten human health [1,2]. Nutrient pollution, such as nitrate and phosphorus, leads to excessive algal reproduction, while organic pollutants lead to the mass reproduction of pathogenic bacteria, viruses, and protozoa that are harmful to human health [3]. Therefore, the prevention and control of water pollution has increasingly become a global concern, and water quality testing is a key link in the treatment of water pollution. At present, the analytical techniques that can be used for water quality detection are mainly based on electrochemical methods [4], spectroscopic methods [5,6,7], and colorimetric methods [8]. While spectroscopic and electrochemical methods offer high detection accuracy, they have significant drawbacks, such as high costs, lengthy processing times, and complex operations, making them unsuitable for field testing. In contrast, colorimetric methods are receiving increasing attention due to their advantages of rapid detection and low consumption. Colorimetric methods are also widely used in water quality testing, where researchers analyze multiple water quality indicators using color signals from specific chromogenic reactions on microfluidic paper-based analysis devices (μPAD) [9,10,11].

With the rapid development of AI (artificial intelligence), new avenues for scientific research have been opened. AI has been widely applied in various fields, including environmental monitoring [12], drug design [13], and materials science [14]. In the area of colorimetric detection, researchers have made significant explorations. For example, Solmaz et al. utilized machine learning classifier algorithms to quantify peroxide content on colorimetric test strips [15]. Dogan et al. employed mobile devices to capture images and conducted their study by cropping regions of interest (ROI). They converted the ROI from the RGB color space to the HSV and Lab color spaces, using the R, G, B, H, S, V, L, a, and b color channels to train machine learning classifiers, achieving a classification accuracy of over 95% [16]. These examples demonstrate the significant success of machine learning in colorimetric analysis; however, traditional machine learning algorithms require manual extraction of data features.

In contrast to traditional image processing and machine learning algorithms, deep learning algorithms can automatically extract image features [17,18,19]. For example, Liu et al. employed two deep learning algorithms and seven machine learning algorithms to assist in colorimetric detection, with a convolutional neural network (CNN) deep learning algorithm performing the best [20]. Recently, Duan et al. utilized deep learning algorithms to automatically extract features in a colorimetric enzyme-linked immunosorbent assay (c-ELISA), avoiding manual feature selection and successfully mitigating the influence of lighting on experimental results [21]. However, most of these studies focus on using deep learning algorithms for classification to output colorimetric information detection results. This approach only achieves single-target detection, thus requiring separate segmentation of regions producing colorimetric signals before applying deep learning algorithms for multi-target detection.

To enhance the versatility of colorimetric detection, object detection algorithms offer possibilities. Treating colorimetric sensors generating different color changes as distinct objects, multi-task detection can be achieved through object detection algorithms. For instance, Chen et al. combined colorimetric methods with the YOLOv3 object detection algorithm, successfully developing a high-precision colorimetric electronic skin for sweat detection [22]. This study validates the feasibility of object detection algorithms and lays the groundwork for multi-task colorimetric detection.

To address the limitations in detection, we propose a multi-task water quality colorimetric detection algorithm based on the YOLOv8n deep learning algorithm. This approach allows for the input of multi-task colorimetric sensor images directly into the deep learning model, achieving fully automated processing from “image input” to “result output”. Unlike traditional methods, our algorithm eliminates the need to segment each task individually for detection.

Firstly, we constructed a comprehensive dataset including images captured under three different light sources and with four different smartphones at various heights. To enhance the dataset’s quality and the algorithm’s robustness, we employed data augmentation techniques for dataset expansion.

Secondly, we made three key improvements upon the YOLOv8n model:

1. We designed the MGFF (Multi-Scale Grouped Feature Fusion) module to replace the c2f module in YOLOv8n. The MGFF module enhances the model’s feature extraction capability while reducing the parameter count through the use of MGConv (Multi-Scale Group Convolution). This approach enables the model to effectively capture and integrate multi-scale features, leading to improved detection performance and efficiency.
2. In the SPPF (Spatial Pyramid Pooling–Fast) module, we introduce LSKA (Large Separable Kernel Attention) to enhance the model’s ability to capture long-range dependencies within the image. This addition helps the model better understand global information and long-range relationships within the image, thereby improving overall detection performance.
3. In the model detection head network, we designed the GNDCDH (Group Norm Detail Convolution Detection Head). In GNDCDH, we introduced shared convolutions to achieve a lightweight design. The combination of Detail Conv (Detail Convolution) and GN (Group Normalization) enhances the model’s performance.

**Figure 1:**
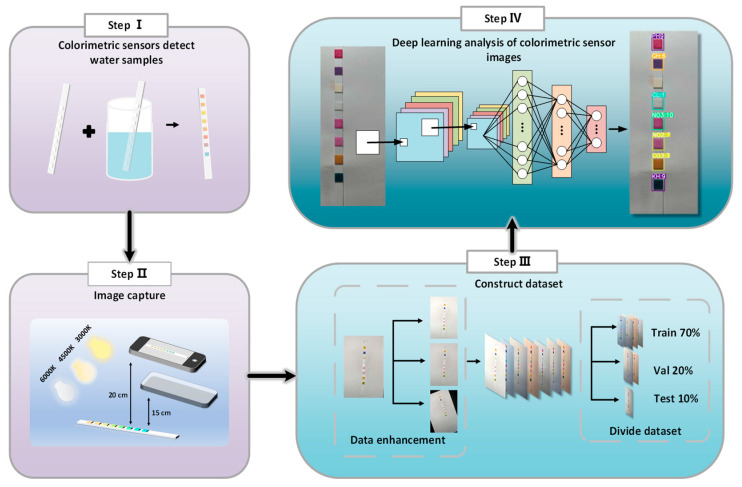
The overview of our method.

Finally, we conducted ablation studies and comparative experiments to comprehensively evaluate the improved model. Compared to seven mainstream deep learning algorithm models, the proposed model demonstrates outstanding performance in accuracy and recall while maintaining the advantage of a low parameter count. These results highlight the effectiveness of the multi-task colorimetric detection algorithm. Figure 1 provides an overview of using deep learning for multi-task colorimetric water quality detection.

## 2. Materials and Methods

### 2.1. Materials

Anhydrous potassium carbonate (Grade: Analytical Reagent), anhydrous calcium chloride (Grade: Analytical Reagent), sodium nitrite (Grade: Analytical Reagent), potassium nitrate (Grade: Analytical Reagent), and sodium hydroxide (Grade: Analytical Reagent) were purchased from Xilong Technology Co., Ltd., Shantou, China. Hydrochloric acid (Grade: Analytical Reagent) was purchased from Chengdu Chron Chemical Co., Ltd., Chengdu, China. Standard solutions for total alkalinity and residual chlorine were purchased from Shanghai Chenwei Technology Co., Ltd., Shanghai, China. The multi-task colorimetric sensor was purchased from Wuhu Jinghui Biotechnology Co., Ltd., Wuhu, China. Deionized water was used throughout the entire research process.

In this study, we selected seven commonly used water quality indicators for detection: pH, hardness, residual chlorine, nitrate, nitrite, carbonate, and total alkalinity. To capture the response of the colorimetric sensor to each indicator at different concentrations, we prepared 11 different concentration solutions for each indicator, with the specific concentration arrangements detailed in Table 1. It is important to note that although the concentration classes may be the same across indicators, these solutions were independently prepared. For example, the pH solution was configured solely based on variations in pH values, while the carbonate solution was adjusted only for carbonate concentration. Solutions for different indicators were prepared independently of one another.

Our multi-task colorimetric sensor is equipped with multiple reaction zones, each de-signed to detect a specific water quality indicator. During the experimental process, we precisely used a micropipette to add each solution at its predetermined concentration to the corresponding reaction zone of the colorimetric sensor. This method was employed to avoid cross-interference between different solutions and to ensure the accurate concentration of solutions in each reaction zone. By employing this precise pipetting technique, we ensured that the solution concentrations in each reaction zone met the preset requirements, thereby enhancing the reliability and accuracy of the experimental data. Finally, we collected image data from the colorimetric sensor for each indicator at varying concentrations, thus constructing the corresponding dataset.

### 2.2. Production of the Dataset

During the image data collection process, various factors, including the optical components of the imaging device, the image sensor, and variations in environmental lighting, may exert differing degrees of influence on the captured images. To ensure that our approach possesses good applicability and robustness, it is essential to construct a comprehensive and diverse dataset that enables the deep learning model to fully learn from these variations and extract meaningful features.

In constructing this dataset, particular attention was given to the impact of lighting conditions and shooting height on the color variations in the colorimetric sensor, in order to better simulate the fluctuations in real-world lighting and height. For example, differences in light sources (such as the type and intensity of the light source) as well as variations in shooting angle and height may lead to color deviations in the image. To address this, three representative light source color temperatures were selected: warm light (3000 K), warm white light (4500 K), and white light (6000 K), to encompass a variety of potential lighting conditions encountered in practical applications.

Additionally, due to the fact that different smartphone brands may employ distinct image processing algorithms, images captured from the same scene may exhibit color differences across devices. Some smartphones tend to enhance color saturation, while others prioritize more natural color reproduction. To minimize the impact of such device-related differences on model predictions, multiple mainstream smartphone brands were used for colorimetric image capture in this study. This approach ensures a more diverse and comprehensive dataset, which facilitates the model’s ability to learn how to handle color variations across different devices and improves its generalization capability across different devices.

Through these measures, we aim to construct a highly representative and adaptable image dataset that will enhance the accuracy and robustness of deep learning algorithms in practical applications.

**Figure 2:**
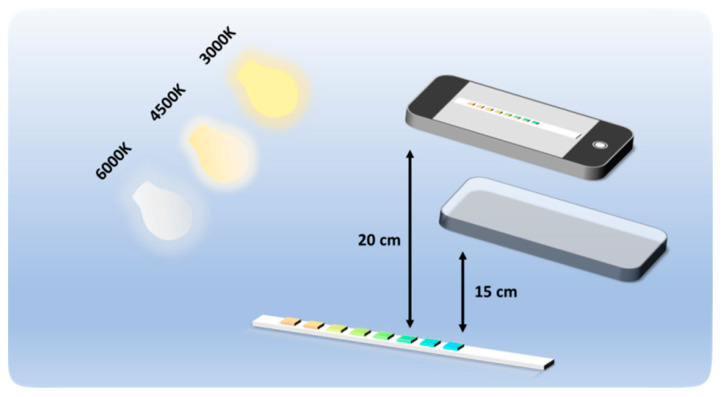
The smartphone photographs the colorimetric sensor.

Four mainstream smartphones are used in this study as shown in Table 2. The smartphone takes colorimetric sensor images from different height angles under different lighting conditions, as shown in Figure 2. The smartphone is set to automatic mode to reduce the influence of human operation.

### 2.3. Data Enhancement

In computer vision, the quality of the dataset plays a crucial role in the performance and generalization ability of models. Data augmentation, as a common technique, is widely used to expand training datasets. In our data augmentation process, we employed various techniques. Specifically, adjusting the contrast of images is an effective method that enhances or diminishes the distinction between different regions of an image. This adjustment helps the model better adapt to various lighting conditions and scene variations. By combining adjustments in brightness and contrast, we generated multiple images to enrich our training dataset.

In addition, we apply common data augmentation methods such as image blurring and noise addition. By fuzzy operation and adding noise, we can further expand the dataset and improve the robustness and generalization ability of the model. These measures work together to improve the performance of the model, so that it achieves a more stable and accurate prediction ability in the face of different situations.

**Figure 3:**
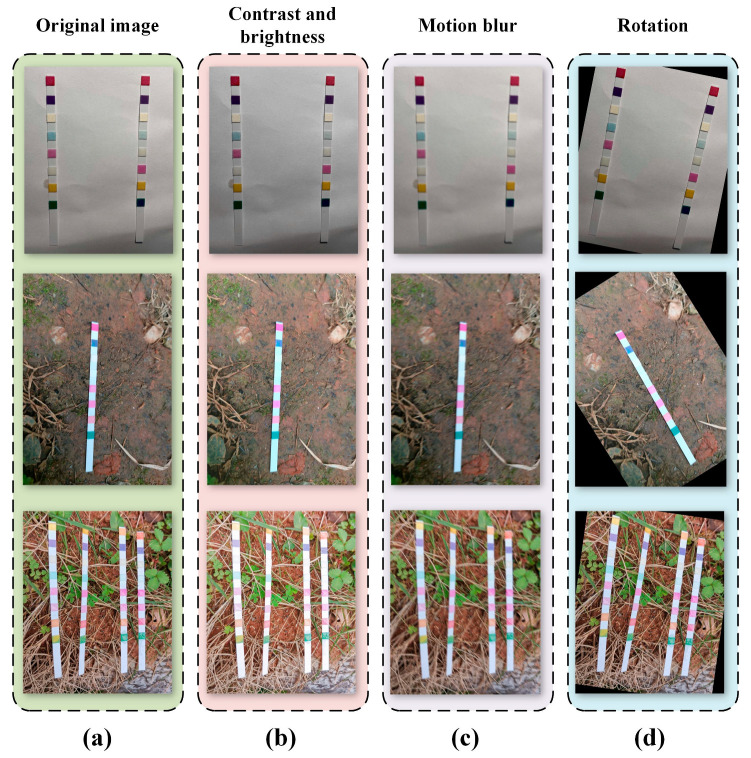
Data enhancement. (**a**) Original image; (**b**) random adjustment of contrast and brightness; (**c**) motion blur; (**d**) random angle rotation.

We employed random adjustments, including contrast, brightness, rotation, and motion blur, to enhance the dataset quality, as illustrated in Figure 3. Following data augmentation, we generated a total of 4500 images. The entire dataset was randomly partitioned into three subsets: a training set, validation set, and test set, at a ratio of 7:2:1. The training set was utilized for model training, while the validation set was employed for fine-tuning model hyperparameters and conducting initial performance assessments. The test set was then employed to evaluate the generalization ability of the final model.

### 2.4. Model Improvement

YOLO is one of the most classic object detection algorithms, and it has been developed into several versions. YOLOv8, developed by Ultralytics, is a versatile model designed for object detection and image segmentation tasks in computer vision. In addition to these core functionalities, YOLOv8 extends its capabilities to include pose estimation, tracking, and classification. YOLOv8 can be applied to the development of almost all visual directions. YOLOv8 combines the advantages of the previous generation of the YOLO series while ensuring the lightweight of the model. Due to its excellent performance, YOLOv8 is widely used in agriculture [23], industry [24], and other fields.

The model architecture of YOLOv8 is structured into three main components: Backbone, Neck, and Head. The Backbone serves as the feature extraction network, responsible for capturing essential information from input images. Positioned between the Backbone and the final output layers, the Neck module enhances the feature representation extracted by the Backbone. The Head component then utilizes these enriched features to generate network predictions.

For water quality colorimetric detection, we proposed three key improvements to the YOLOv8n model. First, we introduced the MGFF module, which is constructed based on MGConv and the Bottleneck module. The MGFF module combines convolution operations at different scales through MGConv, helping the model capture more contextual information while reducing the number of parameters and computational costs.

Next, in SPPF, we introduced the LSKA. This enhancement improves the model’s ability to capture long-range dependencies in images, enabling better understanding of global information and distant relationships within the images. It also mitigates memory consumption and avoids the generation of high computational costs.

**Figure 4:**
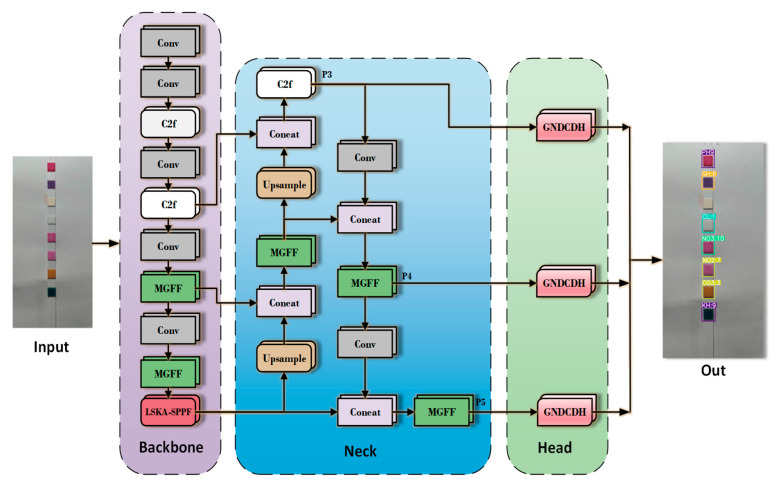
The improved network structure.

Finally, we designed the GNDCDH module. In the GNDCDH, we incorporated shared convolutions, which significantly reduced the number of parameters in the network, effectively decreasing the model complexity and making it more lightweight. Additionally, we included Detail Conv and employed GN to improve the detection accuracy of the detection head. The overall improved network structure is shown in Figure 4.

#### 2.4.1. MGFF Module

**Figure 5:**
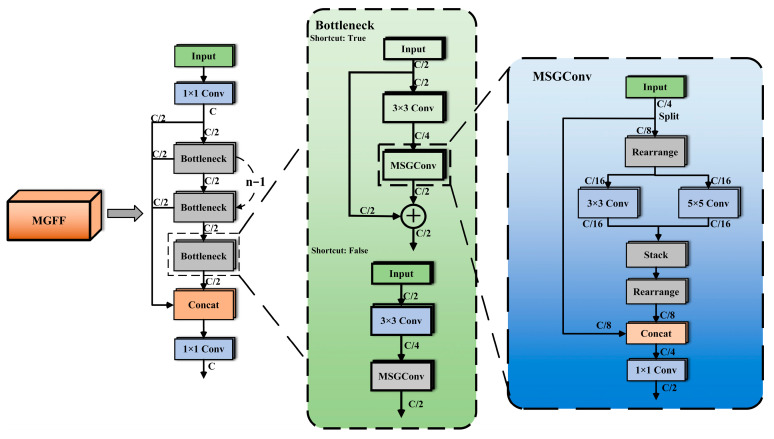
The MGFF module structure.

Inspired by the design philosophy of Bottleneck structures and MGConv, the MGFF module has been developed to replace certain C2f modules in YOLOv8n. The MGFF module consists of several Bottleneck modules, each containing two convolutional layers responsible for transforming the input feature maps to extract high-level features. By employing MGConv to replace some of the standard convolutions within the Bottleneck, the MGFF module is formed. This innovation not only reduces the number of parameters in the network but also improves detection accuracy. The structure of the MGFF module is illustrated in Figure 5, where C represents the number of channels.

MGConv is a simplified convolution module that contains multi-scale information. It draws on the concept of grouped computation to facilitate the extraction of features at different scales. Subsequently, using the point-wise convolution from MobileNet [25], the features of different scales from each group are fused together. In the context of MGConv, the input feature map is divided into two parts, each with a number of channels equal to half of the original feature map’s channels. One part remains unchanged without any convolution operation; meanwhile, the other part performs a high-cost convolution operation. The high-cost convolution operation uses multi-scale convolution, and splits the channel dimension into multiple groups for part of the high-cost convolution calculation. For each group, a convolutional layer is applied separately. The features of each group after operation are concatenated with the parts without any operation in the previous section. Since the feature information is extracted from channels of different sizes and the information within each channel is independent, the features are finally integrated and adjusted by 1 × 1 point-wise convolution. The number of parameters for a standard 3 × 3 convolution can be expressed as follows:

(1) $$Param=(3\times 3\times \frac{C}{4})\times \frac{C}{2}$$

For MGConv, the number of parameters can be represented as:

(2) $$Param=(3\times 3\times \frac{C}{16})\times \frac{C}{16}+(5\times 5\times \frac{C}{16})\times \frac{C}{16}+(1\times 1\times \frac{C}{4})\times \frac{C}{2}$$

Upon calculation, the parameter count for MGConv is approximately 22.9% of that for the 3 × 3 standard convolution in the original network.

The C2f module designed in this paper integrates MGConv to construct a unique network structure. This architecture effectively enhances the model’s ability to represent input features. By incorporating convolution operations across different scales, the model can capture richer context information, which significantly improves the performance and generalization ability of the model. Compared to the standard C2f module in YOLOv8n, the C2f module integrated with MGConv not only reduces the parameter count and computational load but also retains rich feature information.

#### 2.4.2. LSKA-SPPF Module

SPPF is proposed based on SPP (Spatial Pyramid Pooling). SPPF replaces the original three pooling kernels of different sizes of SPP with three 5 × 5 pooling kernels. Different levels of features are acquired by pooling at different scales. Compared with SPP, SPPF significantly reduces the number of parameters, runs faster, and can integrate large-scale global information. This improvement enables SPPF to use fewer resources and achieve more efficient feature extraction and processing while maintaining the advantages of multi-scale feature aggregation.

**Figure 6:**
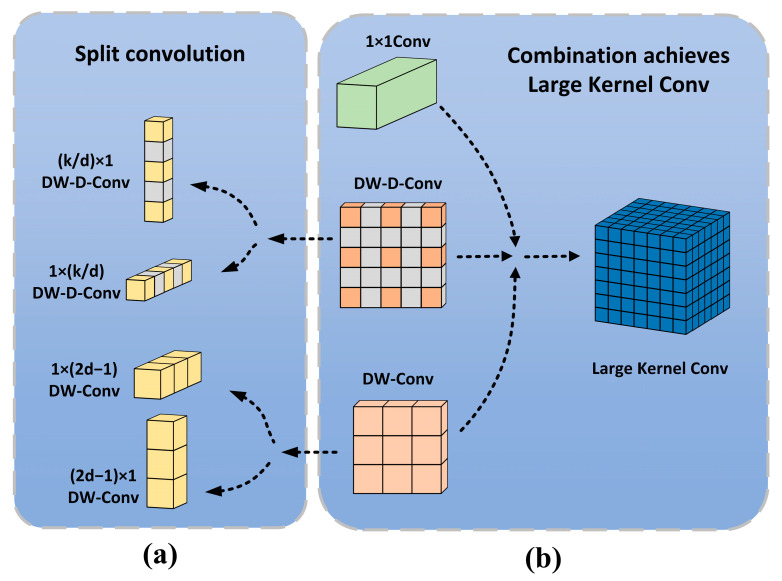
(**a**) Splitting depth-wise convolution and depth-wise dilated convolution; (**b**) combining deep-wise convolution and depth-wise dilated convolution achieves large-scale convolution kernels.

An attention mechanism is a method to strengthen the attention and utilization of important information generated by neural network models. Among them, self-attention is a special form of attention mechanism. It is used to deal with dependencies between elements in sequence data. However, it is worth noting that the self-attention mechanism was originally designed for natural language processing. When it is applied to computer vision tasks, its effect has certain limitations and shortcomings. In a recent study, LKA (Large Kernel Attention) was designed in the VAN [26] (Visual Attention Network) proposed by Guo et al., which realizes adaptation and long-range correlation in self-attention. The LKA module initially employs standard depth-wise convolution with small receptive field kernels to capture local dependencies. Subsequently, it utilizes depth-wise dilated convolution with larger receptive field kernels to capture long-range dependencies. This combination of depth-wise and depth-wise dilated convolutions achieves the effect of large-scale convolution kernels, effectively mitigating the high computational costs associated with using larger convolution kernels in depth-wise convolution operations, as shown in Figure 6b. Lau et al. proposed LSKA [27] based on the LKA module, which split the depth-wise convolution into two separable convolution kernels. The growth in the number of parameters with increasing kernel size is reduced by factoring depth-wise convolutions, and LSKA is computationally efficient even for large kernel sizes. Specifically, a 2D weight kernel by depth-wise convolution and depth-wise dilated convolution is split into two cascaded 1D separable weight kernels, as shown in Figure 6a. The output of LSKA can be expressed mathematically as follows:

(3) $$Y_{1}=\sum_{H}\sum_{W}W_{1\times (2d-1)}*F$$

(4) $$Y_{2}=\sum_{H}\sum_{W}W_{(2d-1)\times 1}*Y_{1}$$

(5) $$D_{1}=\sum_{H}\sum_{W}W_{1\times \left|\frac{k}{d}\right|}*Y_{2}$$

(6) $$D_{2}=\sum_{H}\sum_{W}W_{\left|\frac{k}{d}\right|\times 1}*D_{1}$$

(7) $$A=W_{1\times 1}*D_{2}$$

(8) $$\bar{F}=A⊗F$$

Let F denote the input feature map, where H and W represent its height and width, respectively. The symbol * signifies convolution operation, K represents the maximum receptive field, d denotes the dilation rate, and ⊗ indicates the Hadamard product. Equations (3) and (4) express the output of input features through depth convolutions of size 1 × (2d − 1) and (2d − 1) × 1, and the result is equivalent to the output of depth-wise convolutions of size (2d − 1) × (2d − 1). Similarly, Equations (5) and (6) denote the output of the deep dilated convolution which is split into deep convolutions of $1\times \left|\frac{k}{d}\right|$ and $\left|\frac{k}{d}\right|\times 1$. Equations (3) and (4) depict how the depth-wise convolution captures local spatial information. Equations (5) and (6) illustrate that a depth-wise dilated convolution is employed to capture global spatial information from the output of preceding depth-wise convolutions. The output D_2_ of the depth-wise dilated convolution undergoes a 1 × 1 convolution to derive the attention map A, which is then element-wise multiplied with the input feature map to generate the output feature map.

The growth in the number of parameters as the kernel size increases is reduced by decomposing the depth-wise convolutions, making any of them computationally efficient for larger kernels. The parameters before and after decomposition can be seen as follows:

(9) $$Param={\left(2d-1\right)}^{2}\times C+{\left|\frac{k}{d}\right|}^{2}\times C+C\times C$$

(10) $$Param=(2d-1)\times C\times 2+\left|\frac{k}{d}\right|\times C\times 2+C\times C$$

It can be seen that the parameters in the depth-wise convolution layer are reduced by $\frac{2d-1}{2}$ after decomposition, and the parameters in the depth-wise dilated convolution layer are decreased by $\frac{1}{2}\left|\frac{k}{d}\right|$.

**Figure 7:**
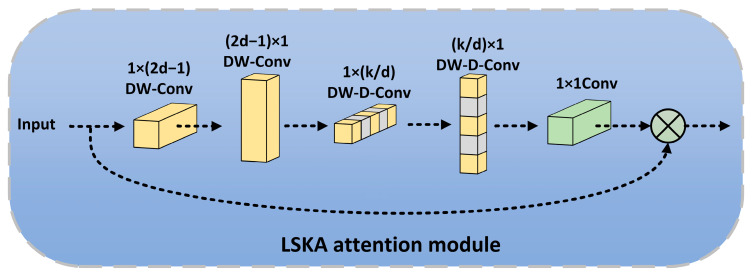
Structural diagram of the LSKA module.

The LSKA module enhances long-range dependencies in input images without incurring high computational costs or excessive memory usage. Compared with general convolution, self-attention, and the LKA module, the LSKA module has four advantages: long-range dependence, spatial and channel adaptability, and scalability to very large kernels. We introduce the LSKA attention module into SPPF to strengthen the ability of SPPF to capture the long-range dependencies in images, help the model better understand the global information and long-range dependencies in images, and thus improve the accuracy and effect of image processing tasks. The structure diagram of the LSKA attention module is shown in Figure 7.

#### 2.4.3. GNDCDH Module

**Figure 8:**
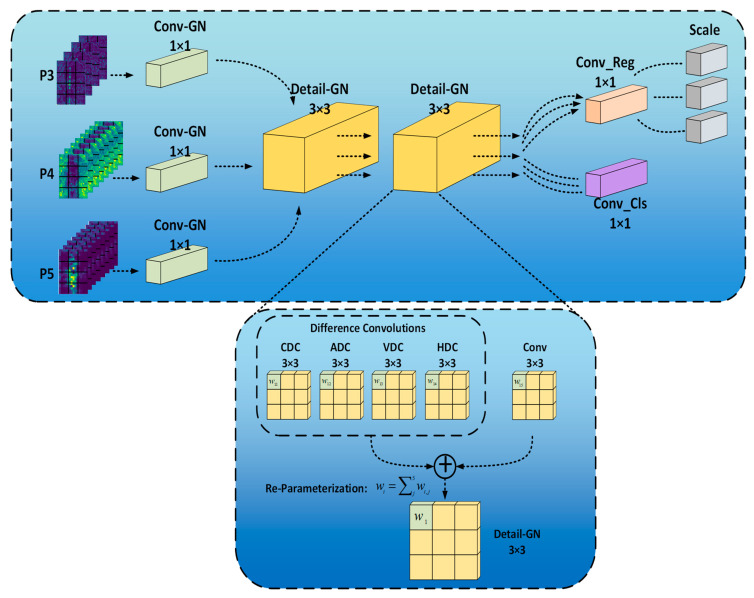
The GNDCDH module structure.

In YOLOv8, the detection head is responsible for final result prediction and output. Compared to YOLOv5, YOLOv8 adopts a decoupled head structure in its detection head, while YOLOv5 uses a coupled head. The decoupled head structure divides the detection head into two branches: classification (Cls) and bounding box regression (Box), which leads to a significant increase in the number of parameters. The substantial increase in parameters is detrimental to deploying the model on resource-constrained embedded devices. To facilitate future model deployment, a lightweight detection head has been designed. We named this detection head the Group Normalization Detail Convolution Detection Head (GNDCDH). In the GNDCDH, we incorporate shared convolutions. The use of shared convolutions allows for parameter sharing between different layers, thereby reducing the overall model size and effectively decreasing model complexity, making the model more lightweight. Due to the lightweighting, there may be a reduction in the network’s ability to extract features. To compensate for the performance loss caused by lightweighting, we introduced Detail Conv [28] and employed Group Normalization. Detail Conv enhances the ability to capture details, improving the detection accuracy of the detection head. In the detection head network, Group Normalization is applied to Detail Conv to enhance the performance of the detection head in terms of localization and classification. These innovations collectively contribute to a more efficient detection head, balancing a lightweight architecture with high performance. The GNDCDH structure is shown in Figure 8.

The three outputs from the Neck network serve as inputs to the detection head. To achieve lightweighting, the input feature maps are processed through 1 × 1 convolutions. Compared to 3 × 3 convolutions, 1 × 1 convolutions have a smaller computational load, effectively reducing the overall computational burden. The outputs of the 1 × 1 convolutions are then used as inputs for the Detail Convolution. The Detail Convolution consists of difference convolutions and a standard convolution. The differential convolutions include Central Difference Convolution (CDC), Angle Difference Convolution (ADC), Horizontal Difference Convolution (HDC), and Vertical Difference Convolution (VDC). The standard convolution is used to capture intensity-level information, while the differential convolutions focus on gradient-level information. The standard convolution and differential convolutions are deployed in parallel for feature extraction. Through reparameterization techniques, the Detail Conv is equivalently transformed into standard convolution without introducing additional parameters or computational costs, thereby enhancing efficiency. The outputs of two sequential Detail Convolutions are fed into convolutional layers that generate bounding box coordinates and class probabilities. To maintain lightweighting, the Detail Convolutions, along with the convolutional layers for generating bounding box coordinates and class probabilities, are designed as shared convolutions.

It is worth noting that, to compensate for the performance loss caused by lightweighting, we use GN (Group Normalization) in the detection head network. In both the 1 × 1 convolutions and Detail Convolutions, we replace BN (Batch Normalization) with GN. GN divides the channels into groups and calculates the mean and variance within each group for normalization. The computation of GN is independent of the batch size, and its accuracy remains stable across various batch sizes. It has been demonstrated in FCOS [29] that GN can enhance the localization and classification performance of the detection head.

## 3. Results and Discussion

### 3.1. Experimental Environment Configuration

The experiments were conducted on a Windows 11 system, with Python version 3.9 and CUDA version 11.8. The training and testing were performed using the PyTorch 2.0.1 deep learning framework. The hardware environment of the experimental platform includes an AMD Ryzen 7 6800H CPU and an NVIDIA GeForce RTX 3070 Ti GPU.

### 3.2. Evaluation Metrics

The evaluation indicators for deep learning algorithms can be mainly divided into two categories: detection accuracy and model complexity. Detection accuracy is primarily reflected by the model’s precision (P), recall (R), average precision (AP), and mean average precision (mAP).

If the sample is positive, and it is predicted to be positive, this is called a true positive (TP); If the sample is positive, but it is predicted as negative, this is called a false negative (FN). If a sample is negative, but it is predicted as positive, this is called a false positive (FP). If the sample is a negative example and is predicted as a negative example, this is called the true negative class (TN).

P is defined as the proportion of the number of samples that are actually positive examples and predicted to be positive examples to the total number of samples predicted to be positive examples, as follows:

(11) $$P=\frac{TP}{TP+FP}.$$

R is defined as the proportion of samples that are actually positive examples and predicted to be positive examples to the total number of samples that are actually positive examples, as follows:

(12) $$R=\frac{TP}{TP+FN}.$$

The AP method is not used to calculate the average precision. The AP evaluates the performance of the model by calculating the area of the PR curve (precision–recall curve) for each category and the axis. This area can be calculated by means of an integral. The larger the value of AP, the larger the area surrounded by the PR curve and coordinate axes; that is, the precision and recall are relatively high on the whole.

mAP is used to evaluate the detection accuracy of the model in each category. mAP is the average of AP values over all categories. AP can reflect the accuracy of each category’s prediction, and mAP is the average of the AP of all categories to reflect the accuracy of the whole model. The higher the mAP value, the better the detection ability of the model. mAP50 and mAP95 are commonly used metrics for evaluating the accuracy of object detection models. mAP50 refers to mAP calculated using a fixed intersection over union (IOU) threshold of 50%. IOU measures the overlap between a predicted bounding box and a ground truth bounding box. A prediction is considered correct if the IoU is greater than or equal to 50%, indicating that at least 50% of the predicted and ground truth bounding boxes overlap. mAP50 represents the average precision of the model across all categories and detections at this threshold. In contrast, mAP95 is a more stringent evaluation metric. It computes mAP across multiple IOU thresholds, typically ranging from 50% to 95%, with increments of 5%. The final mAP95 score is the average of the mAP values calculated at these varying IOU thresholds. mAP95 provides a more comprehensive assessment of the model’s performance, as it accounts not only for easier cases where a lower IOU threshold is acceptable, but also for more challenging cases that require a higher IOU threshold to consider a prediction correct.

Giga Floating Point Operations (GFLOPs) is a commonly used metric to quantify the computational complexity of deep learning models, representing one billion floating-point operations. In the context of deep learning, particularly during the training and inference phases of neural networks, floating-point operations are a critical aspect of the overall computation. By calculating the GFLOPs count of a model, one can estimate the computational requirements for both inference and training tasks, providing valuable insights into the model’s performance demands and hardware requirements.

### 3.3. Ablation Experiment

Ablation experiments were conducted to verify the importance of the MGFF, LSKA-SPPF, and GNDCDH modules in achieving good model performance. This helps to better understand the impact of each module on the overall model performance and the contribution of each module. The evaluation was conducted using five metrics: P, R, mAP, Parameters, and GFLOPs.

Table 3 demonstrates that Models 1–7 outperform the original YOLOv8n across various metrics. In Model 2, the P improved by 1.7%, indicating that the MGFF module enhances the model’s capability to capture features at different scales. In Model 3, the parameter count was reduced by 23%, while both P and R increased, suggesting that the GNDCDH module exhibits exceptional performance by effectively leveraging Detail Convolution and Group Normalization to capture finer details as well as improve localization and classification. In Model 4, the feature extraction capability of MGFF, combined with the feature fusion capability of LSAK-SPPF, resulted in an increase in P of 2.3% and R of 2.9%. Model 5 achieved the lowest parameter and computational costs among all models while still exceeding the original YOLOv8n in all metrics, indicating the successful lightweight design of the MGFF and GNDCDH modules. In Model 6, the combination of LSAK-SPPF and GNDCDH led to a 2% increase in P and a 1.4% increase in mAP50. These experiments further validate the significance of the MGFF, LSAK-SPPF, and GNDCDH modules in enhancing model performance. Model 7 demonstrated the best overall performance, driven by the synergistic effects of the three modules: P increased by 2.8%, R improved by 3.5%, mAP50 rose by 2%, and mAP95 increased by 1.9%, with a reduction in parameter count of 25.8% and computational cost by 25.6%. The aforementioned ablation studies provide compelling evidence of the importance of the MGFF, LSAK-SPPF, and GNDCDH modules in achieving a lightweight design and enhancing model performance.

### 3.4. Comparison Experiment

In this study, a series of improvements were made to the original YOLOv8n model with the aim of enhancing accuracy and efficiency in water quality colorimetric detection. To validate the overall performance of the proposed model, comprehensive comparative experiments were conducted against mainstream two-stage and one-stage object detection algorithms. The performance of the model introduced in this research was thoroughly evaluated. The comparative experiments utilized the same dataset for training and evaluation, adhering to the principle of controlled variables, with consistent hardware and software conditions throughout the experimental process.

As shown in Table 4, the parameter count and computational load of Faster R-CNN are significantly higher than those of the other models, and its performance is considerably inferior. YOLOv8s and YOLOv8m demonstrate commendable results in P, R, and mAP; however, their high parameter counts and computational demands render them unsuitable for deployment on embedded devices. Although YOLOv9-t achieves favorable P, it exhibits relatively low R, indicating a higher rate of missed detections. YOLOv5n has the smallest parameter count and computational load among the models assessed, yet its performance is also subpar.

In contrast, YOLOv8n and YOLOv10n strike a relative balance between performance and model size; however, YOLOv8n outperforms YOLOv10n in P, R, and mAP. Compared to YOLOv8n, the proposed model reduces the parameter count by 25.8% and computational load by 25.6%, with all performance metrics surpassing those of YOLOv8n. Our model achieves performance comparable to YOLOv8s, while its parameter count is merely 20.5% of that of YOLOv8s and its computational load is 22.4% of YOLOv8s.

### 3.5. Results Analysis

**Figure 9:**
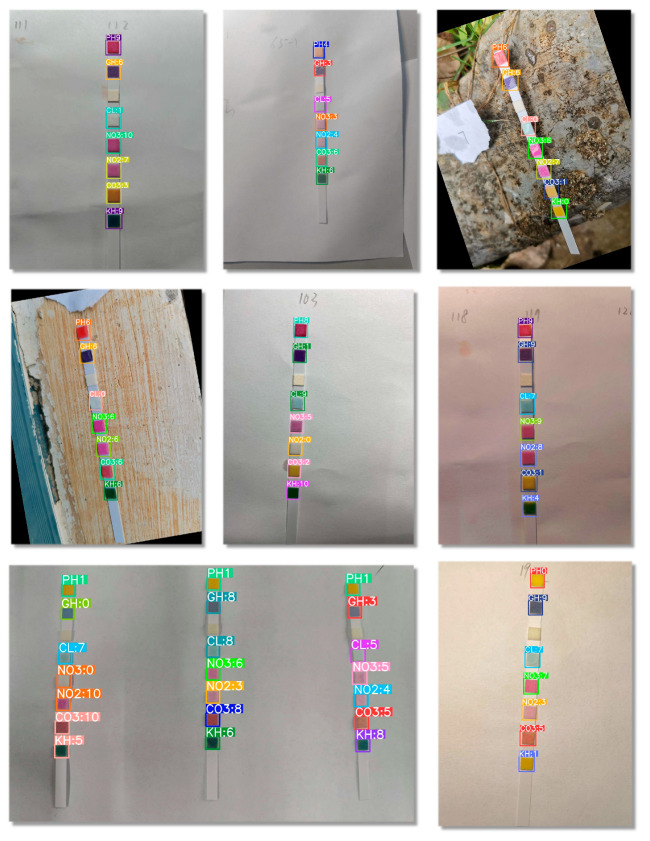
Model detection results in different scenarios.

In this study, the improved model was validated for the colorimetric detection of water quality in different scenarios. The detection results are compared in a relatively fixed indoor environment and in an outdoor complex background. Figure 9 shows the detection results of our model.

Evaluating the effectiveness of models is crucial, and visualization offers a more intuitive perspective for this assessment. It allows for the observation of detected object locations, sizes, and categories, which are essential for evaluating model accuracy and efficiency, as well as for debugging and optimization purposes. By employing visualization techniques, we conducted a comparative analysis of the model’s performance before and after improvements. We visualized the model’s predictions using statistical methods, highlighting correct predictions with green bounding boxes, incorrect predictions with blue bounding boxes, and undetected objects with red bounding boxes.

**Figure 10:**
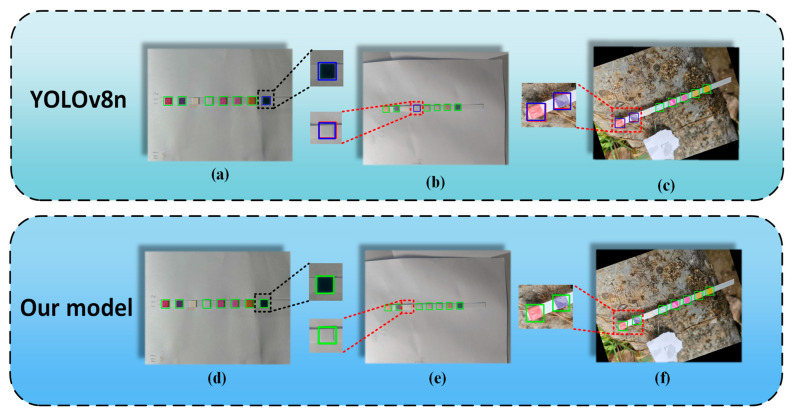
Visual statistical results.

Figure 10 displays several visualization results, where (a–c) correspond to the outcomes from YOLOv8n, and (d–f) represent results from our proposed model. In the results from YOLOv8n, the last detection region on the right side of the first image is indicated by a blue bounding box, signifying an erroneous prediction by the model in that area. Conversely, our proposed model correctly identifies the outcome in the same location, as depicted in Figure 10d. In Figure 10b, a red bounding box overlaps with a blue bounding box in the central reaction area of the colorimetric sensor. Based on the visualization statistics, this overlap indicates that YOLOv8n experiences both false positives and missed detections for this target. This phenomenon is also observed in Figure 10c with the YOLOv8n model. In contrast, as shown in Figure 10e,f, our proposed model successfully detects the results, while YOLOv8n exhibits both missed detections and false positives in these areas.

This research demonstrates that our proposed model significantly alleviates issues related to false negatives and false positives compared to the original model, thereby validating its effectiveness in colorimetric water quality detection tasks.

In this work, we employed heatmap visualization techniques to identify which areas of the image significantly influence the prediction results. Grad-CAM [30] is a classic method for interpreting convolutional neural networks (CNNs). However, due to the side effects of the gradient averaging step in Grad-CAM, it sometimes highlights locations that the model does not actually utilize. To address this limitation, Rechel et al. proposed a novel method for interpreting CNNs called HiResCAM [31]. HiResCAM is a gradient-based interpretation technique that generates a class-specific attention map for each category, thereby emphasizing the parts of the input image that contribute to specific predictions. This method mitigates the issues present in Grad-CAM by exclusively highlighting the locations used by the model for each prediction. By leveraging HiResCAM, we can better elucidate the internal workings of convolutional neural networks, enhancing their interpretability and reliability.

**Figure 11:**
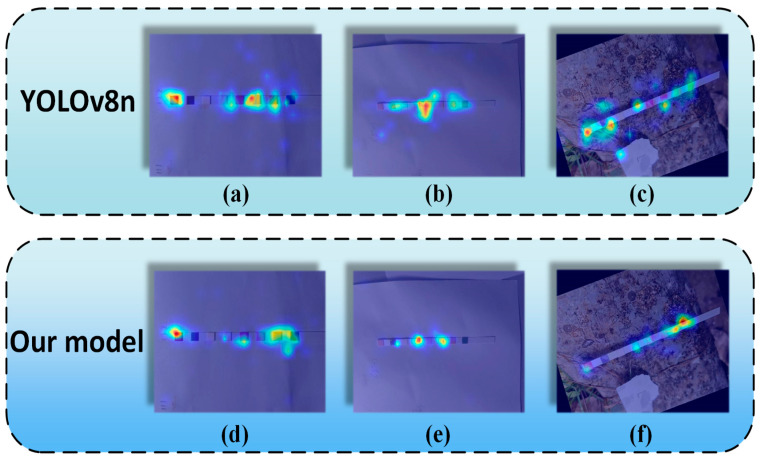
The comparison of the HiResCAM heatmaps between YOLOv8n and the model proposed in this paper.

The heatmaps generated by HiResCAM reflect the contribution of various elements in the image to the model’s prediction results. Figure 11 presents several outcomes. In the heatmaps, regions where the network model allocates more attention are shown in red, indicating a higher contribution to the network’s prediction results, while areas in blue signify a lower contribution. A comparison between Figure 11a,d reveals that the improved model focuses more on the color sensor. In contrast, the attention distribution of the YOLOv8n model, as illustrated in Figure 11b,e, is relatively dispersed, whereas the proposed model concentrates its attention on the regions where the YOLOv8n model makes errors. Figure 11c,f demonstrate that although the YOLOv8n model allocates significant attention to the left side, it still produces incorrect predictions. In contrast, the improved model achieves correct predictions with less attention. Additionally, it is evident that the proposed model places greater emphasis on regions with similar colors.

In our study, we conducted a thorough analysis of the model’s prediction errors. The analysis revealed that the majority of prediction errors occurred in situations involving high solution concentrations, particularly when approaching the maximum detection limit of the colorimetric sensor. We hypothesize that the primary reason for this phenomenon lies in the non-linear relationship between the color change intensity of the sensor and the solution concentration. As the concentration approaches the sensor’s maximum detection range, the color change tends to saturate, leading to a significant reduction in the magnitude of color variations. These subtle changes pose a considerable challenge for the model in predicting high-concentration solutions accurately.

To address this issue, future research could focus on enhancing the model’s ability to learn subtle changes when approaching the maximum detection concentration of the colorimetric sensor. Additionally, the dataset could be augmented with additional samples in the high-concentration range, which would enable the model to better learn and capture these minute variations.

## 4. Conclusions

This study presents a multi-task colorimetric detection model for water quality based on YOLOv8n. By utilizing multi-task colorimetric sensors and deep learning models, we achieve fully automated processing from “image input to result output”. We designed the MGFF module, which effectively enhances the model’s feature extraction capability while reducing the overall number of parameters by integrating feature information of different sizes. To capture long-range dependencies within the images, we introduced LSKA into SPPF. This integration facilitates the model’s improved understanding of the global information and long-range relationships present in the images. Within the model’s detection head network, we developed the GNDCDH module, which improves detection accuracy while maintaining a lightweight architecture.

Ablation experiments were conducted to validate the effectiveness of our proposed improvements. We compared our model against seven mainstream deep learning algorithms. The results indicate that the enhanced model achieves outstanding performance while maintaining a low parameter count. Specifically, precision and recall at mAP50 reached 96.4%, 96.2%, and 98.3%, respectively. Compared to the YOLOv8n model, we reduced the parameter count by 25.8% and the computational load by 25.6%, while simultaneously improving detection P by 2.8%, R by 3.5%, mAP50 by 2%, and mAP95 by 1.9%.

In summary, our findings demonstrate the effectiveness of the deep learning-based multi-task colorimetric detection approach for water quality assessment, providing a robust tool for rapid on-site water quality monitoring. Future research will focus on further enhancing P, R, and mAP, as well as deploying the model on embedded devices for practical applications.

## Figures and Tables

**Table 1:** Solution concentrations.

Solution	Class 1	Class 2	Class 3	Class 4	Class 5	Class 6	Class 7	Class 8	Class 9	Class 10	Class 11
PH	5.2	5.6	6.0	6.4	6.8	7.2	7.6	8.0	8.4	8.8	9.2
Hardness (mg/L)	0	100	200	300	500	400	600	700	800	900	1000
Residual chlorine (mg/L)	0	0.5	1.0	1.5	2.0	2.5	3.0	3.5	4.0	4.5	5.0
Nitrate (mg/L)	0	25	50	75	100	125	150	175	200	225	250
Nitrite (mg/L)	0	1	2	3	4	5	6	7	8	9	10
Carbonate (mg/L)	0	20	40	60	80	100	120	140	160	180	200
Total alkalinity (mg/L)	0	20	40	60	80	100	120	140	160	180	200

**Table 2:** Smartphone camera properties.

Smartphone	Aperture	Pixels (Million)	Sensor Size
iPhone 12	f/1.6	12	1/1.76
OnePlus Ace Pro PGP110	f/1.8	50	1/1.56
HUAWEI nova4e	f/1.8	24	1/2.8
Redmi k50 ultra	f/1.6	108	1/1.67

**Table 3:** Ablation experiment results.

Model	MGFF	LSKA-SPPF	GNDCDH	P (%)	R (%)	mAP50 (%)	mAP95 (%)	Parameters (Million)	GFLOPs
YOLOv8n				93.6	92.7	96.3	63.4	3.1	8.6
Model1	√			95.3	94.6	97.5	64.3	2.8	8.3
Model2		√		95.5	94.2	97.8	64.6	3.4	8.8
Model3			√	95.1	95.7	97.7	64.1	2.4	6.6
Mode4	√	√		95.9	95.6	98.0	64.5	3.1	8.4
Model5	√		√	95.6	95.1	97.7	64.4	2.1	6.1
Model6		√	√	95.6	95.3	97.7	64.5	2.6	6.8
Model7	√	√	√	96.4	96.2	98.3	65.3	2.3	6.4

**Table 4:** Comparison experiment results.

Model	P (%)	R (%)	mAP50 (%)	Parameters (Million)	GFLOPs
Faster R-CNN	49.3	89.3	72.0	130.1	370.2
YOLOv5n	88.5	83.1	88.9	1.9	4.5
YOLOv8n	93.6	92.7	96.3	3.1	8.6
YOLOv8s	96.3	96.1	98.3	11.2	28.6
YOLOv8m	96.5	95.9	98.2	25.9	78.9
YOLOv9-t	95.0	90.2	95.5	3.7	16.2
YOLOv10n	92.9	90.5	96.0	2.8	8.5
Our model	96.4	96.2	98.3	2.3	6.4

## Data Availability

The data presented in this study are available from Shaojie Wu upon request.
